# Force-field functor theory: classical force-fields which reproduce equilibrium quantum distributions

**DOI:** 10.3389/fchem.2013.00026

**Published:** 2013-10-25

**Authors:** Ryan Babbush, John Parkhill, Alán Aspuru-Guzik

**Affiliations:** ^1^Department of Chemistry and Chemical Biology, Harvard UniversityCambridge, MA, USA; ^2^Department of Chemistry, The University of Notre DameSouth Bend, IN, USA

**Keywords:** effective potentials, path integral molecular dynamics, nuclear quantum propagation, liquid hydrogen, density functional theory

## Abstract

Feynman and Hibbs were the first to variationally determine an effective potential whose associated classical canonical ensemble approximates the exact quantum partition function. We examine the existence of a map between the local potential and an effective classical potential which matches the *exact* quantum equilibrium density and partition function. The usefulness of such a mapping rests in its ability to readily improve Born-Oppenheimer potentials for use with classical sampling. We show that such a map is unique and must exist. To explore the feasibility of using this result to improve classical molecular mechanics, we numerically produce a map from a library of randomly generated one-dimensional potential/effective potential pairs then evaluate its performance on independent test problems. We also apply the map to simulate liquid *para*-hydrogen, finding that the resulting radial pair distribution functions agree well with path integral Monte Carlo simulations. The surprising accessibility and transferability of the technique suggest a quantitative route to adapting Born-Oppenheimer potentials, with a motivation similar in spirit to the powerful ideas and approximations of density functional theory.

## Introduction

The energy and mass scales of chemical motion lie in a regime between quantum and classical mechanics but for reasons of computational complexity, molecular modeling (MM) is largely performed according to Newton's laws. When classical Hamiltonians are chosen to reproduce properties of real material, classical MM is an efficient compromise. An increasing amount of MM uses highly accurate Born-Oppenheimer (BO) potential energy surfaces, which allow one to study complex bond rearrangements where experiment cannot motivate a potential (Car and Parrinello, 1985; Wang et al., 2013). The BO surface is incompatible with classical statistical mechanics in the sense that we would not expect a classical simulation on the BO surface to reproduce properties of the real material, except in the limit of infinite temperature.

Many approaches already exist to bridge this gap and study quantum equilibrium properties and dynamics: path integral Monte Carlo (PIMC), ring polymer molecular dynamics (RPMD), centroid molecular dynamics (CMD), variational path-integral approximations, discretized path-integral approximations, semi-classical approximations, thermal Gaussian molecular dynamics and colored-noise thermostats (Whitlock et al., 1979; Chandler and Wolynes, 1981; Jang et al., 2001; Nakayama and Makri, 2003; Poulsen et al., 2003; Liu and Miller, 2006; Paesani et al., 2006; Fanourgakis et al., 2009; Liu et al., 2009; Ceriotti et al., 2011; Georgescu et al., 2011; Pérez and Tuckerman, 2011). Most of the these methods involve computational overhead significantly beyond classical mechanics and as they approach exactness their cost rapidly increases.

An alternative philosophy is suggested by density functional theory (DFT) (Hohenberg and Kohn, 1964; Mermin, 1965; Sham and Kohn, 1966; Runge and Gross, 1984; Yuen-Zhou et al., 2010; Tempel and Aspuru-Guzik, 2011). Following this line of reasoning, three questions arise. Can an equilibrium quantum density be obtained from purely classical mechanics and an effective Hamiltonian? Is the effective Hamiltonian uniquely determined by the physical potential? Can the particle density and free energy be given by such a fictitious system? To all these questions, the answer “yes” is implied by the usual recipe for classical force-fields that fit experimental data. The idea of using an effective classical Hamiltonian to incorperate nuclear quantum propagation effects is not novel. For the first time, we prove the uniqueness and existence of a map yielding a classical effective potential given the physical potential. We also make a contribution to this field by demonstrating that the aforementioned mapping can be reversed numerically and approximated analytically.

The bargain of our proposed effective classical potential is similar to that posed by DFT. One sacrifices access to rigorous momentum based-observables and abandons the route to systematic improvement. In exchange, the two properties which are physically guaranteed, the equilibrium particle density and the partition function, are obtained at a cost equivalent to classical sampling but with improved accuracy. As a practical tool, the map is an easy way to transform BO-based force fields into a form which is well-suited for classical sampling. Perhaps the most promising aspect of this mapping would be its scalability which could potentially extend the ability to treat quantum propagation effects to all systems that can be sampled classically. It is even possible that the fictitious trajectories of particles moving on such a potential would, like Kohn-Sham orbitals, have somewhat improved physicality over their classical counterparts, although we will not examine that possibility here.

First, we show the uniqueness of an equilibrium effective potential that gives the exact equilibrium quantum density via classical sampling. Next, we demonstrate that the equilibrium effective potential may be approximated by a linear operator acting on the true potential. Finally, we numerically approximate the map in a rudimentary way, and obtain surprisingly good results and transferability for both one dimensional potentials and a model of liquid *para*-hydrogen.

## 1. Equilibrium effective potential

In their seminal work on path integral quantum mechanics, Feynman and Hibbs introduced the concept of an effective classical potential that allows for the calculation of quantum partition functions in a seemingly classical fashion (Feynman and Hibbs, 1965). In Appendix A, we discuss a connection with the large and fruitful body of research that focuses on the centroid effective potential and density which should not be confused with the equilibrium effective potential that we now define (Giachetti and Tognetti, 1985; Feynman and Kleinert, 1986; Voth, 1991; Cao, 1993, 1994a,b,c; Cuccoli et al., 1995; Cao and Martyna, 1996; Martyna, 1996; Pavese and Voth, 1996; Roy et al., 1999; Krajewski and Muser, 2001; Blinov and Roy, 2004; Hone et al., 2005; Roy, 2005; Mielke et al., 2013). We start by considering the equilibrium density matrix,

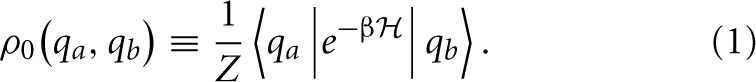



where 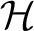 is the system Hamiltonian, β is the inverse temperature, and *Z* is the partition function. Feynman showed us that we could connect this expression to the path integral representation of the quantum propagator^1^,

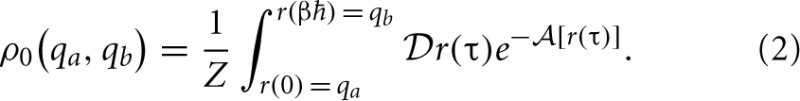



where the Wick-rotated (*t* → −*i*τ) action functional is,

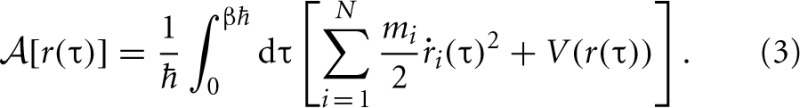



By integrating over only closed paths at each coordinate we obtain the scalar equilibrium density,

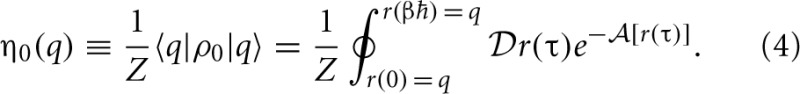



Finally, we define the partition function as a normalization factor which is obtained by integrating over *q*,

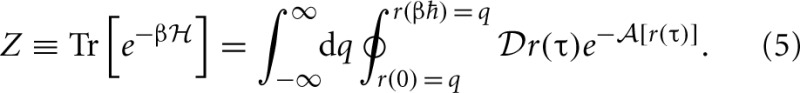



We are now in a position to define an equilibrium effective potential, which encapsulates knowledge of the physical quantum density into a form amenable to classical sampling. We choose the equilibrium effective potential, *W*(*q*) such that,

(6) $$\eta_{0}(q)\equiv \frac{1}{Z}e^{-\beta W(q)}$$

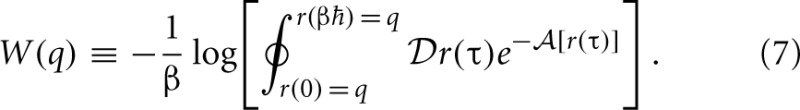

Note that this definition associates the Boltzmann factor, *e*^−β*W*(*q*)^, with the *unnormalized* density. Because η_0_(*q*) must integrate to unity, this allows us to easily recover the partition function and corresponding quantum Helmholtz free energy, *A*, with the classical integral,

(8) $$\int_{-\infty }^{\infty }\text{d}q\;e^{-\beta W(q)}=Z\int_{-\infty }^{\infty }\text{d}q\;\eta_{0}(q)=Z\equiv e^{-\beta A}.$$

**Figure 1:**
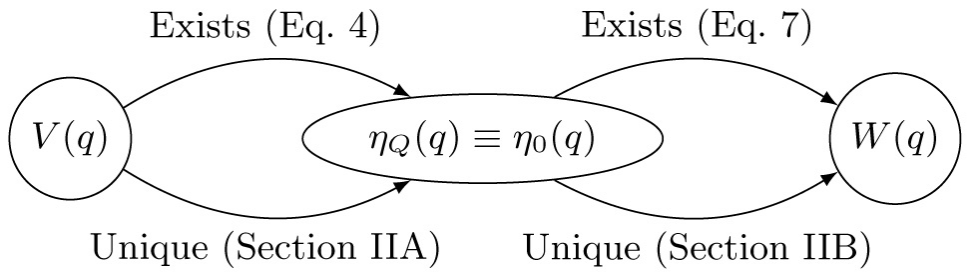
**Morphism depicting uniqueness and existence of mappings between the physical potential, *V*(*q*), the equilibrium effective potential, *W*(*q*), and the associated quantum and classical equilibrium densities, η_*Q*_(*q*) and η_0_(*q*), respectively.** This establishes the existence of a mapping, 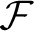, which uniquely determines the equilibrium effective potential.

Using Equation 7, one can exactly calculate the equilibrium effective potential whenever one can evaluate the path integral. Unfortunately that is usually numerically intractable. Thus, it is useful to wonder if a *unique* map exists between any potential *V*(*q*) and *W*(*q*) under the conditions of a fixed ensemble. If one could easily evaluate the map one could transferably adapt BO potentials to give physical results in classical simulations. Since this mapping is a functor^2^ which gives an effective force-field we refer to the map as the “force-field functor” and denote it with the symbol 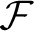. A morphism depicting the structure of our proof is shown in Figure 1.

## 2. Uniqueness and existence

Our first step toward developing a theory of force-field functors is to show that the proposed mapping, 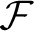 [*V*(*q*)] → *W*(*q*), exists and is unique. This proof begins in Part A of the current section in which we argue that no two *V*(*q*) lead to the same quantum equilibrium density η_0_(*q*), which exists by Equations 3 and 4. To show this we take inspiration from Mermin's extension of the Hohenberg-Kohn theorem for finite temperatures and use the quantum Bogoliubov inequality to construct a proof by contradiction (Mermin, 1965). For any potential without hard-shell interactions, the density is always given by a Boltzmann factor of the potential as in Equation 6; thus, the equilibrium effective potential exists for any physically-relevant quantum potential. In Part B of the current section, we make a similar argument to prove that there is a one-to-one map between classical equilibrium density and classical potential (Chayes et al., 1984). Since the effective potential is chosen to be the classical potential associated with the quantum density, these results directly imply that the map between physical potential and effective potential must be unique.

### 2.1. Uniqueness of quantum density

Both steps in this proof take the form of *reductio ad absurdum* arguments based on the uniqueness of an ensemble which minimizes the free energy of a canonical system. In the Appendix B we show that,

(9) $$A[\rho ]\;>A\left[\rho_{0}\right],\;\rho \neq \rho_{0}$$

where *A* is the quantum Helmholtz free energy,

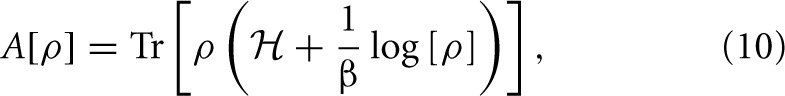



which is minimum when ρ is equal to the quantum equilibrium density matrix ρ_0_ associated with the Hamiltonian, 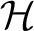 = *T* + *V*(*q*). With this in mind, suppose that there were another potential $\tilde{V}(q)$ that led to the same density η_0_(*q*). Denote the Hamiltonian, canonical density matrix and Helmholtz free energy associated with $\tilde{V}(q)$ by 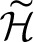, ${\tilde{\rho }}_{0}$, and $\tilde{A}$. Since $\tilde{V}(q)\neq V(q)$ and $\tilde{\rho_{0}}\neq \rho_{0}$^3^. we can write

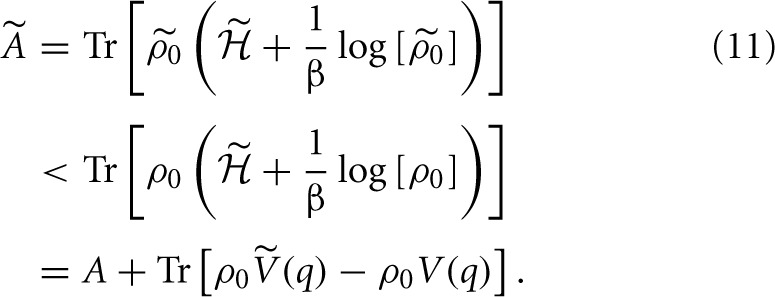



Using the definition of the quantum equilibrium particle density,^4^

(12) $$\eta_{0}(q)\equiv \text{Tr}\left[\rho_{0}|q\rangle \;\langle q|\right],$$

we see that,

(13) $$\tilde{A}<A+\int \text{d}q\left[\tilde{V}(q)-V(q)\right]\eta_{0}(q).$$

But we see that this relation is still true if we interchange over-scored variables,

(14) $$A<\tilde{A}+\int \text{d}q\left[V(q)-\tilde{V}(q)\right]\eta_{0}(q).$$

This leads to the contradiction,

(15) $$A+\tilde{A}<\tilde{A}+A.$$

and therefore only one *V*(*q*) can result in a given η_0_(*q*). This proves that *V*(*q*) uniquely determines η_0_(*q*). Next, we show that the only potential which can reproduce the quantum density with classical sampling is the equilibrium effective potential.

### 2.2. Uniqueness of the effective potential

Equation 7 shows the existence the equilibrium effective potential, *W*(*q*). It remains to be shown that *W*(*q*) is the only such potential which will reproduce the quantum density, which is to say that 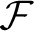 is completely unique. The classical Bogoliubov inequality states that,

(16) $$A\left[\tilde{\eta_{0}}(q)\right]>A\left[\eta_{0}(q)\right],\;\tilde{\eta_{0}}(q)\neq \eta_{0}(q)$$

where *A* is the classical Helmholtz free energy,

(17) $$\begin{array}{l} A\left[\eta_{0}(q)\right]=E\left[\eta_{0}(q)\right]-\frac{1}{\beta }S\left[\eta_{0}(q)\right]\\ \;=\int \text{d}q\;\eta_{0}(q)W(q)+\frac{1}{\beta }\int \text{d}q\;\eta_{0}(q)\log\left[\eta_{0}(q)\right] \end{array}$$

which is minimum when η_0_(*r*) is equal to the classical equilibrium density in the presence of *W*(*q*). For completeness, this result is also proved in Appendix C. With this in mind, suppose that there were two effective potentials, $\tilde{W}(q)$ and *W*(*q*) that led to the same density. Then,

(18) $$\begin{array}{l} \tilde{A}=\int \text{d}q\;\eta_{0}(q)\tilde{W}(q)+\frac{1}{\beta }\int \text{d}q\;\eta_{0}(q)\log\left[\eta_{0}(q)\right]\\ \;<\int \text{d}q\;\eta_{0}(q)W(q)+\frac{1}{\beta }\int \text{d}q\;\eta_{0}(q)\log\left[\eta_{0}(q)\right]\\ \;=A+\int \text{d}q\;\eta_{0}(q)\left[\tilde{W}(q)-W(q)\right]. \end{array}$$

So we see that,

(19) $$\tilde{A}<A+\int \text{d}q\;\eta_{0}(q)\left[\tilde{W}(q)-W(q)\right].$$

If we interchanged all over-scored quantities, we would also find the following,

(20) $$A<\tilde{A}+\int \text{d}q\;\eta_{0}(q)\text{​}\left[W(q)-\tilde{W}(q)\right].$$

Adding these equations together leads to the result,

(21) $$\tilde{A}+A<A+\tilde{A}.$$

Thus, we see that no two *W*(*q*) lead to the same η_0_(*q*).

Because the physical potential *V*(*q*) uniquely determines the quantum equilibrium density η_0_(*q*), and the quantum equilibrium density uniquely determines the equilibrium effective potential *W*(*q*), we see that the map, 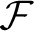
[*V*(*q*)] → *W*(*q*) must be completely unique.

## 3. Approximate linearity

The results of the above section establish the possibility of reversing 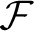 by modeling pairs of *V*(*q*) and *W*(*q*) generated via the exact path-integral. However, the concept of 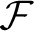 is not useful unless we have good reason to suspect that 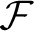 or a useful approximation to 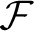 will be easy to obtain and evaluate numerically. In this section, we analyze the approximation of 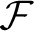 as a linear functor which is straightforwards to construct numerically and because of its separability, applicable to systems of arbitrary dimensionality.

We begin by rewriting Equations 4 and 6,

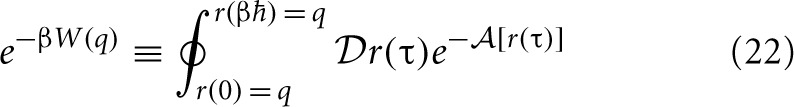



and introduce several definitions which break apart the action term into a kinetic part and a potential part,

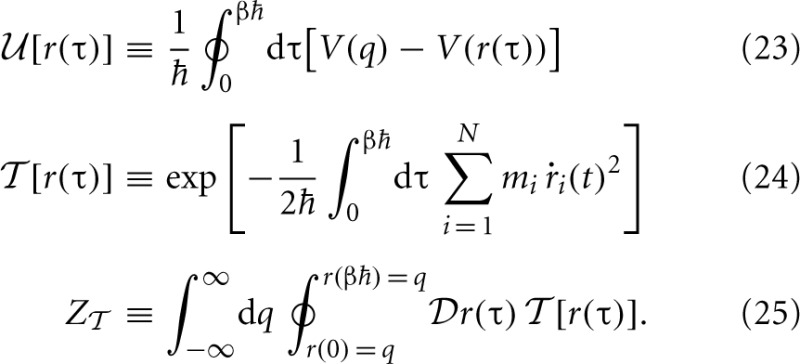



We now employ a notation due to Feynman and Hibbs, for the equilibrium average of a path functional weighted by 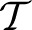 and normalized by 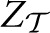, “〈〉” (Feynman and Hibbs, 1965). This allows us to write a concise, exact expression for *W*(*q*):

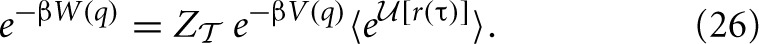



Jensen's inequality tells us that that, 〈*e*^*f*^〉 ≥ *e*^〈*f*〉^ with an error on the order of the variance of *f*. This simplifies the path integral and introduces error that is second order at worst in the weighted path functional average,

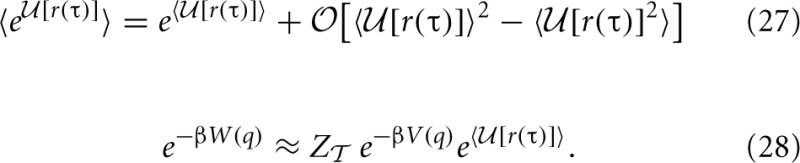



Because any potential is unique only up to a constant, we can use properties of logarithms to remove 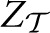, since it does not depend on *q* or *V*(*q*), to write

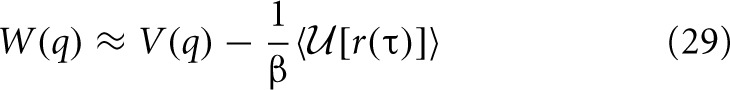



with corrections on the order of 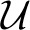
^2^. We also see from this that the equilibrium effective potential is a temperature dependent correction to the true potential. 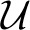
[*r*(τ)] is clearly a linear functional of *V*(*q*) and 〈
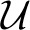
[*r*(τ)]〉 is clearly a linear functor of 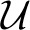
[*r*(τ)],

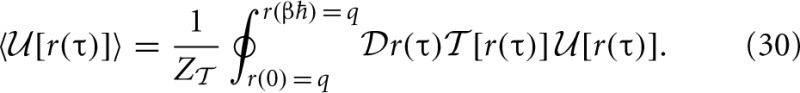



In the multi-dimensional case, the path integral couples all 3*N* modes of *q*, making the exact 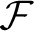 a very complicated object which embeds all-orders of quantum many body effects between these modes. However, our analysis suggests a linear approximation which conserves the locality of the original potential. With this approximation we can separate the integral in Equation 30 into each individual interaction order of the potential and see that the path integral does not multiply these terms; the pairwise interactions remain pairwise, the three-mode interactions are mapped by 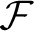 onto three-mode interactions, etc.

Approximate separability of this mapping is one of the key differences between our method and approaches such as Feynman-Kleinert, which introduces higher ordered many-body terms into the effective potential, or RPMD, which avoids the issue at the cost of introducing ancilla particles. Our 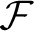 can be imagined as a Gaussian smearing of *V*(*q*) to first approximation. It is reasonable to suspect that the non-separable many body couplings would be blurred to a high order such that the many-body expansion of the equilibrium effective potential might terminate faster than the many-body expansion of the uncorrected physical potential. This agrees with the empirical observation that tunneling effects stabilize pairwise interactions more than higher-ordered interactions.

## 4. Numerical tests

It is far from obvious that a transferable map between *V*(*q*) and *W*(*q*) can be practically obtained and usefully accurate. Instead of calling upon the most sophisticated procedures we can implement to solve the problem, we take *the simplest* approach to developing and testing our approximation to 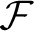 so that our results are designed to be a worst-case, upper-bound on the error that leaves room for optimism. Approaches such as machine learning could be employed in future work (Snyder et al., 2012). We approximate 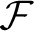 as a linear map (a matrix) acting on our potentials vectorized into coefficients of Legendre polynomials. The entries of this matrix are determined by simple least-squares on a randomly generated training set of 1000 one-dimensional potentials and their corresponding effective potentials chosen by randomly choosing Legendre coefficients with the only constraint being that the classical densities vanish at their boundaries.

Effective potentials were calculated using Equation 7 with densities obtained from the efficient real-space discrete variable representation (DVR) of the path integral (Thirumalai et al., 1983). We examine how this 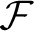 performs on instances of other random potentials not included within its training set and then apply it to the Silvera-Goldman pair potential for liquid *para*-hydrogen (Silvera and Goldman, 1978; Nakayama and Makri, 2003; Hone and Voth, 2004; Poulsen et al., 2004; Miller and Manolopoulos, 2005). Using the resulting effective potential, a classical Monte Carlo simulation was performed to give us radial pair distribution functions in agreement with results from PIMC at a fraction of the computational cost.

### 4.1. Obtaining the linear functor

In order to obtain the simplest possible 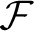 we model the linear transformation as a matrix. This requires that we treat the physical potential and effective potential as vectors in some basis of real-valued functions. Because force-fields are often chosen for the speed with which they can be evaluated, it seems natural to use a polynomial basis. Legendre polynomials evaluated on a fixed domain of [−1, 1] were chosen for their orthogonality and historical usefulness in fitting potentials.

Consider the short time Trotterization of the path-integral, which we use to generate exact quantum densities for our test sets (Thirumalai et al., 1983). The short-time propagator effectively acts as a Gaussian which blurs out the density with a variance that depends exactly on the inverse of the square root of the the mass times the temperature. This factor which determines the “quantumness” of the system is proportional to the thermal de Broglie wavelength, $\Lambda =\hbar \sqrt{2\pi \beta /m}$ (Yonetani and Kinugawa, 2003; Georgescu et al., 2013). Because we wish to calculate the deformation of a potential vector evaluated on a fixed domain, the parameter which characterizes our map must depend on the ratio between the thermal de Broglie wavelength and the potential length-scale, *Q* = Λ/*L* where *L* is the potential length-scale.

In order to obtain a linear functor capable of transforming a one-dimensional potential at fixed *Q* into another one-dimensional potential at fixed *Q* we randomly generated pairs of potential vectors and their corresponding effective potential vectors. These vectors were in a Legendre polynomial basis of order *B* and the vector elements of the classical potential (i.e., basis coefficients) were drawn from a flat distribution between −10/β and 10/β. The corresponding effective potential vectors were calculated by evaluating the classical potential vectors as Legendre polynomials on the fixed domain, passing the scalar potential and *Q* to the aforementioned DVR routine which yields a scalar quantum density, and finally fitting the negative logarithm of that density divided by β to a vector of Legendre polynomials in accordance with Equation 7. Having done this, the goal is to find a matrix 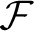 ∈ *B* × *B* such that, 
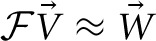
. We chose to perform a Levenberg-Marquart L2 optimization to determine the elements of this matrix (Levenberg, 1944). Our residual was defined as the concatenation of the difference vectors, 
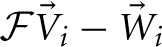
 for all *N* physical potential / effective potential pairs in the randomly generated set.

### 4.2. Performance analysis

**Figure 2:**
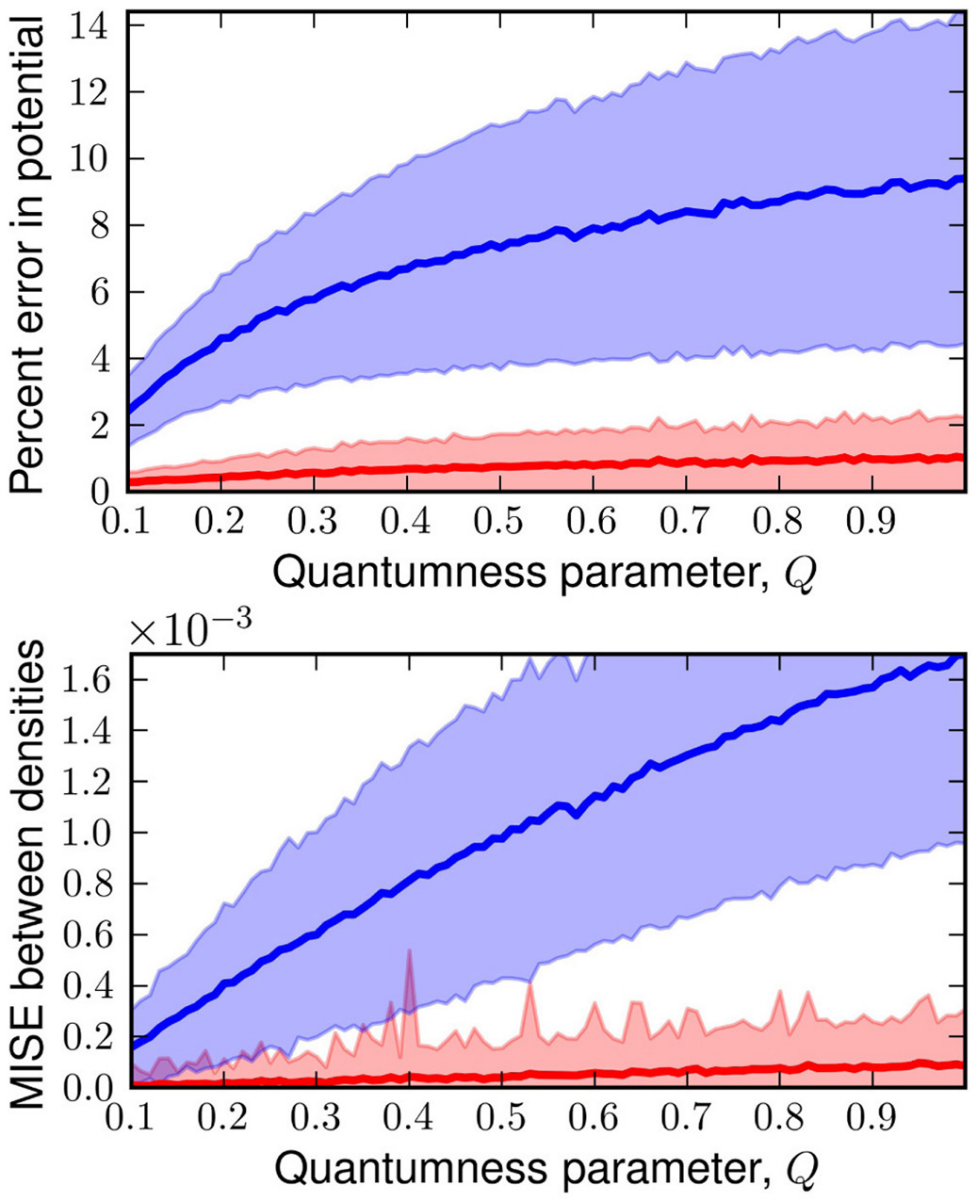
**Top:** plot of the percent error in potential energy of a classical simulation with the classical potential (blue) and 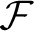 generated distribution (red) against *Q*. **Bottom:** plot of mean integrated squared error (MISE) from the exact quantum density for classical density (blue) and 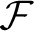 generated density (red) against *Q*. Each point is the mean of these errors on 1000 random potentials with 50 basis functions.

**Figure 3:**
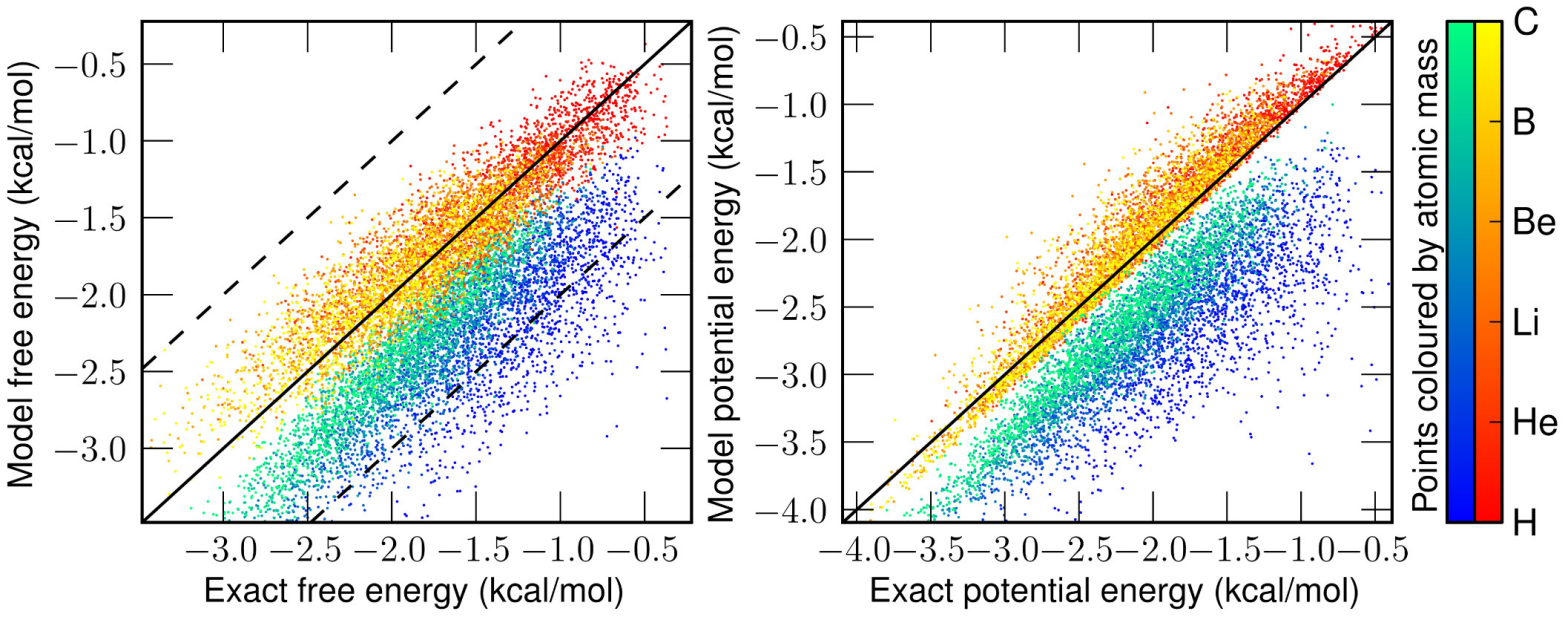
**Left:** correlation of classical (blue-green) and 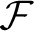 generated (red-yellow) free energy with exact free energy. Dotted lines enclose the chemically accurate region of within one kcal/mol. *In more than 97% of instances*, our map is more accurate than the classical treatment. **Right:** correlation of classical and 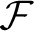 generated potential energy with exact potential energy. Color brightness indicates the mass used in setting the *Q* value at 25K. As mass increases, classical simulations better approximate the energy. Data consists of 1000 cross-validating potentials at each of the six masses shown on the colorbar.

The linear approximation to 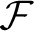 appears to work quite well for even fairly large values of *Q*. As we can see in Figure 2, the errors on an independent test set from the linear 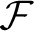 generated *W*(*q*) are minimal and significantly better than the classical predictions, especially in strongly quantum regimes. Even the deviation from the exact answer is improved relative to simulations which employ the uncorrected physical potential. For both simulations the error goes to zero as *Q* goes to zero—a consequence of classical correspondence. As one might expect as *Q* is increased, predictions given by both classical and 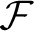 generated distributions deviate more significantly from the exact answer. In the *W*(*q*) simulations these errors are entirely due to the linearity of 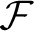. Another view of the the performance of the linear functor is given in Figure 3. When temperature and length are fixed, mass is a reasonable predictor of the performance of both *W*(*q*) and *V*(*q*) simulations. For low masses, the classical treatment often misses the quantum free energy by as much as a kcal/mol (chemical accuracy). Having characterized the error of assuming linearity we turn to separability.

**Figure 4:**
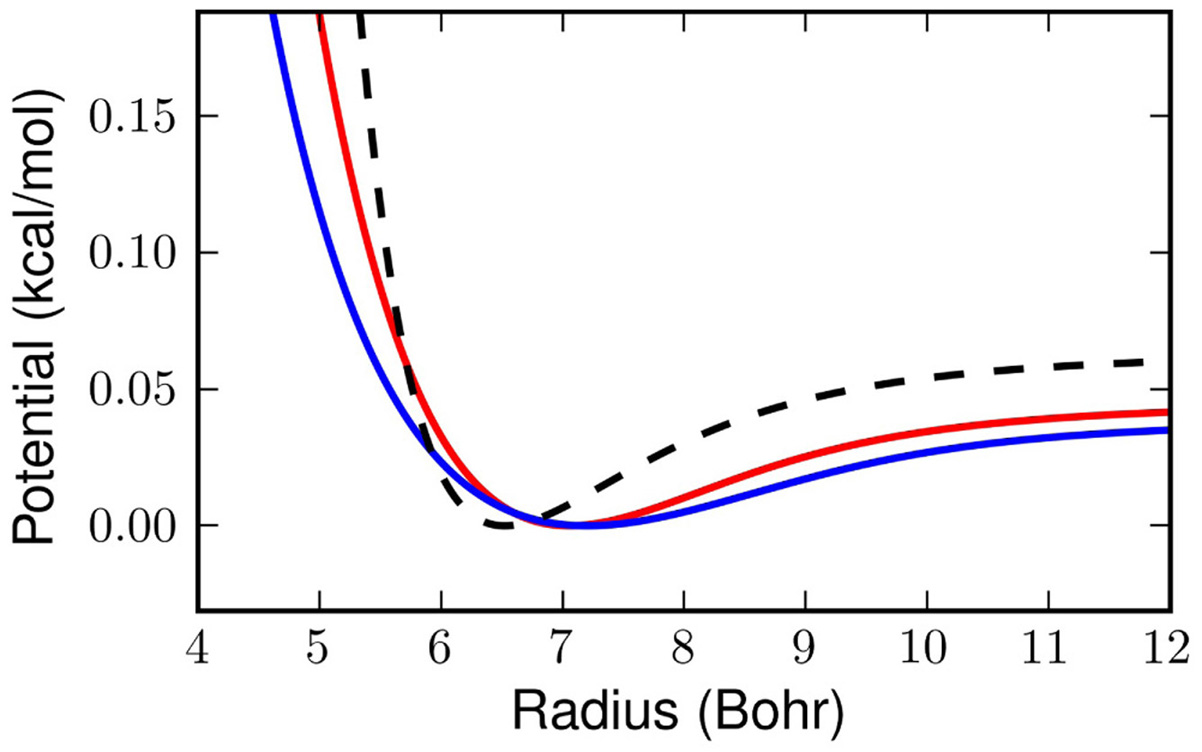
**The dashed black line above shows the classical Silvera-Goldman potential in the region of interest for our problem.** The red line is the effective potential obtained with our linear 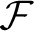 at 25K and the blue line is at 14K.

Figure 4 shows the effective potentials obtained from applying our linear 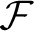, trained at 14K and 25L with sets of 1000 potentials, to the Silvera-Goldman potential, which is perhaps the most common potential used to simulate liquid hydrogen with path integrals (Silvera and Goldman, 1978; Nakayama and Makri, 2003; Hone and Voth, 2004; Poulsen et al., 2004; Miller and Manolopoulos, 2005). We then performed a classical Monte Carlo simulation on the potential mapped at 25K and the potential mapped at 14K, using 150 molecules in a cubic cell with periodic boundary conditions and one million steps. Cell size was fixed by densities from the literature (Nakayama and Makri, 2003).

**Figure 5:**
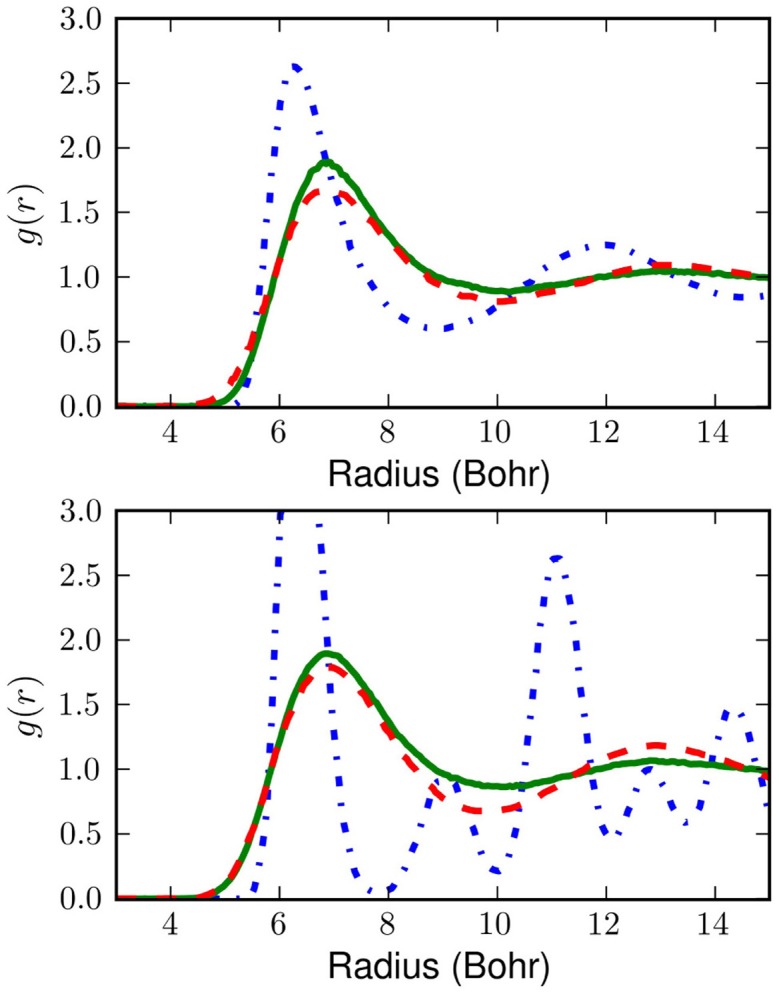
**The top box shows radial pair distribution functions at 25K and the bottom box shows radial pair distribution functions at 14K.** The blue (dotted-dashed) curve is for the classical liquid without correction for quantum effects. The green curve (solid) shows the result of classical Monte Carlo sampling on the effective potential obtained with our linear 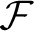. The red curve (dashed) shows PIMC results (Nakayama and Makri, 2003). Even this simple 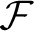 is a major improvement over the classical potential.

The resulting radial distribution functions, *g*(*r*) are shown in Figure 5. The differences between the *W*(*q*) generated *g*(*r*) and the PIMC results are presumably due to the assumption of separability. Slight over-structuring of *g*(*r*) at the first shell results from neglect of the 3-body components of the exact *W*(*q*). Remarkably, this over-structuring appears to decrease with temperature, lending credence to the idea that many-body effects in *W*(*q*) are largely blurred-out by the smearing which the low orders of 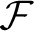 perform on the potential. At both temperatures the errors of these approximations are quite reasonable and although the classical system undergoes a non-physical transition to a solid between 25 and 14K, the model of the present work remains correct.

## 5. Conclusion

We have shown that for each physical potential, there is a unique effective potential which reproduces the quantum density and free energy when sampled with classical statistics. Other properties of a classical simulation of the effective Hamiltonian are not designed to approximate reality by the mapping, but the effective potential may be advantageous to the status quo: classical simulation on a Born-Oppenheimer surface. In this paper we have shown that the implied mapping between the physical and effective potential, 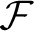, can be made concrete to a useful degree of accuracy. A simple linear model for 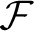 improves on the physical potential systematically over a broad range conditions. Even under the assumption of separability and without any exponential functions in our training set, our model for 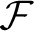 adequately describes the density of a popular *para*-hydrogen model at exactly the cost of the corresponding classical simulation. Non-linear models for 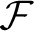 and expressions which do not assume complete separability are likely to improve on these results and produce even more accurate transferable recipes for digesting Born-Oppenheimer potentials. In particular, we imagine the development of simple functors which could be applied to Born-Oppenheimer surfaces so that classical sampling will immediately give improved results. Ultimately, we believe that force-field functors can provide a scalable methodology for including quantum propagation effects in systems that are intractable for exact methods, such as protein force-fields.

## Author contributions

All authors conceived and designed the research project. With guidance from John Parkhill and Alán Aspuru-Guzik, Ryan Babbush wrote the proofs, first draft of the manuscript, and code for obtaining and characterizing the numerical functor. All authors interpreted the results and co-wrote the article.

### Conflict of interest statement

The authors declare that the research was conducted in the absence of any commercial or financial relationships that could be construed as a potential conflict of interest.

## Footnotes

^1^Throughout this paper the variable “*q*” refers to a position in the full coordinate space of the system (*q* ∈ ℜ^3*N*^ where *N* is the number of particles). To distinguish a position variable from a path variable we will use *r*(*t*) to represent a particular trajectory.

^2^A functor differs from a functional in that a functor maps one vector space to another whereas a functional maps a vector space to a scalar. In this context, “operator” is a more common term than “functor” but we prefer to call this “force-field functor theory” to evoke the connection with DFT.

^3^That the corresponding equilibrium density matrices are not equal is obvious in Equation 1

^4^Recall that *q* ∈ ℜ^3*N*^ so, |*q*〉 = ∏^3*N*^_*i* = 1_ |*q*_*i*_〉.

## A1. Appendix

### A1.1. Quantum densities from classical sampling

In practice, path integral expressions are analytically intractable except in a few cases. Feynman proposed to simplify Equation 5 by changing from an integral over all closed paths that start and end at point *q* to an integral over all closed paths that have an average value equal to the path centroid *r*,

(A1) $$\bar{r}=\frac{1}{\beta \hbar }\int_{0}^{\beta \hbar }\text{d}\tau \;r(\tau ).$$

So that we only integrate over each closed path once, we must change our expression for the partition function to only calculate paths that match the centroid,

While the partition functions given by Equation 5 and Equation A2 are exactly equal, the two expressions are associated with subtly different scalar density functions. Equation 5 is associated with the true equilibrium density in Equation 4 and A2 is associated with the path centroid density,

The Dirac delta function in this equation enforces the requirement that integrating the Boltzmann factor associated with this density over the path centroid, *r*, will result in exactly the path integral expression for the quantum partition function (Jang and Voth, 1999). The centroid density plays a prominent role in CMD and Feynman-Kleinert methods but does not apply to force-field functor theory.

### A1.2. Proof of quantum bogoliubov inequality

The quantum Bogoliubov inequality is proved for the grand canonical ensemble in the Appendix of (Mermin, 1965). We adapt this proof for the canonical ensemble, in the interest of completeness, to show that for all positive definite ρ with unit trace,

(A4) $$A[\rho ]>A\left[\rho_{0}\right],\;\rho \neq \rho_{0}$$

if *A* is the quantum Helmholtz free energy of the canonical ensemble,

which is minimum only when ρ is equal to the quantum equilibrium density matrix ρ_0_ associated with the Hamiltonian,  = *T* + *V*(*q*). To start we define,

where,

We see that ρ_λ_ = ρ_0_ if λ = 0 and ρ_λ_ = ρ if λ = 1. Accordingly,

(A8) $$A[\rho ]-A\left[\rho_{0}\right]=\int_{0}^{1}\frac{\partial }{\partial \lambda }A\left[\rho_{\lambda }\right]\text{d}\lambda$$

by the fundamental theorem of calculus. To evaluate the derivative we use,

The first trace is stationary for variations of ρ_λ_ about the corresponding density matrix. Thus, we only need to differentiate the second trace,

(A10) $$\frac{\partial }{\partial \lambda }A\left[\rho_{\lambda }\right]=-\lambda \text{Tr}\left[\Delta \frac{\partial }{\partial \lambda }\rho_{\lambda }\right].$$

We evaluate $\frac{\partial }{\partial \lambda }\rho_{\lambda }$ using the operator identity,

(A13) $$\frac{\partial }{\partial \lambda }\rho_{\lambda }=-\int_{0}^{\beta }\text{d}\beta^{\prime }\rho_{\lambda }\left[\Delta_{\lambda }\left(\beta^{\prime }\right)-{\langle \Delta \rangle }_{\lambda }\right],$$

where

(A14) $${\langle X\rangle }_{\lambda }=\text{Tr}\left[\rho_{\lambda }X\right].$$

Therefore,

(A15) $$\frac{\partial }{\partial \lambda }A\left[\rho_{\lambda }\right]=\lambda \int_{0}^{\beta }\text{d}\beta^{\prime }\left({\langle \Delta \Delta_{\lambda }\left(\beta^{\prime }\right)\rangle }_{\lambda }-{\langle \Delta \rangle }_{\lambda }^{2}\right).$$

By cyclically permuting operators within the trace, one can verify that

(A16) $${\langle \Delta_{\lambda }\left(\beta^{\prime }\right)\rangle }_{\lambda }={\langle \Delta \rangle }_{\lambda }\;\forall \beta^{\prime },$$

(A17) $${\langle \Delta \Delta_{\lambda }\left(\beta^{\prime }\right)\rangle }_{\lambda }=\langle \Delta_{\lambda }{\left(\frac{1}{2}\beta^{\prime }\right)}^{†}\Delta_{\lambda }\left(\frac{1}{2}\beta^{\prime }\right)\rangle .$$

With these identities, we can rewrite Equation A15,

(A18) $$\frac{\partial }{\partial \lambda }A\left[\rho_{\lambda }\right]=\lambda \int_{0}^{\beta }\text{d}\beta^{\prime }{\langle {\left(\Delta_{\lambda }\left(\frac{1}{2}\beta^{\prime }\right)-{\langle \Delta \rangle }_{\lambda }\right)}^{†}\left(\Delta_{\lambda }\left(\frac{1}{2}\beta^{\prime }\right)-{\langle \Delta \rangle }_{\lambda }\right)\rangle }_{\lambda }.$$

This integral is non-negative and can be zero only if Δ is a multiple of the unit operator, i.e., if ρ_0_ = ρ. This proves that the minimum of the free energy must occur when ρ_λ_ = ρ_0_.

### A1.3. Proof of classical bogoliubov inequality

If η_0_(*q*) is the equilibrium density for a classical canonical ensemble and $\tilde{\eta_{0}}(q)$ is a different density, Gibbs' classical Bogoliubov inequality states that,

(A19) $$A\left[\tilde{\eta_{0}}(q)\right]>A\left[\eta_{0}(q)\right],\;\tilde{\eta_{0}}(q)\neq \eta_{0}(q)$$

where *A* is the classical Helmholtz free energy,

(A20) $$\begin{array}{l} A\left[\eta_{0}(q)\right]=E\left[\eta_{0}(q)\right]-\frac{1}{\beta }S\left[\eta_{0}(q)\right]\\ \;=\int \text{d}q\;\eta_{0}(q)W(q)+\frac{1}{\beta }\int \text{d}q\;\eta_{0}(q)\log\left[\eta_{0}(q)\right]. \end{array}$$

To see that this is the case we start by writing,

(A21) $$\frac{1}{\beta }\int \text{d}q\;\tilde{\eta_{0}}(q)\log\left[\tilde{\eta_{0}}(q)\right]\geq \frac{1}{\beta }\int \text{d}q\;\tilde{\eta_{0}}(q)\log\left[\eta_{0}(q)\right].$$

The difference between the right and left sides of this equation is,

(A22) $$\frac{1}{\beta }\int \text{d}q\left(\tilde{\eta_{0}}(q)\log\left[\tilde{\eta_{0}}(q)\right]-\tilde{\eta_{0}}(q)\log\left[\eta_{0}(q)\right]\right)=\frac{1}{\beta }\int \text{d}q\;\tilde{\eta_{0}}(q)\log\left[\frac{\tilde{\eta_{0}}(q)}{\eta_{0}(q)}\right].$$

Because $\log\left[x\right]\geq 1-\frac{1}{x}$ and we know that the densities are normalized,

(A23) $$\frac{1}{\beta }\int \text{d}q\;\tilde{\eta_{0}}(q)\log\left[\frac{\tilde{\eta_{0}}(q)}{\eta_{0}(q)}\right]\geq \frac{1}{\beta }\int \text{d}q\left[\tilde{\eta_{0}}(q)-\eta_{0}(q)\right]=0.$$

We can simplify this further to,

(A24) $$\langle \frac{1}{\beta }\log\left[\eta_{0}(q)\right]\rangle \geq \langle \frac{1}{\beta }\log\left[\tilde{\eta_{0}}(q)\right]\rangle .$$

We know that,

(A25) $$\eta_{0}(q)=\frac{e^{-\beta E(q)}}{Z}$$

(A26) $$\tilde{\eta_{0}}(q)=\frac{e^{-\beta \tilde{E}(q)}}{\tilde{Z}}$$

where $\tilde{E}(q)$ and $\tilde{Z}$ correspond to the energy and partition function associated with $\tilde{\eta_{0}}(q)$. Thus,

(A27) $$\langle \frac{1}{\beta }\log\left[\frac{e^{-\beta \tilde{E}(q)}}{\tilde{Z}}\right]\rangle \geq \langle \frac{1}{\beta }\log\left[\frac{e^{-\beta E(q)}}{Z}\right]\rangle$$

(A28) $$\langle -\tilde{E}(q)-\frac{1}{\beta }\log\left[\tilde{Z}\right]\rangle \geq \langle -E(q)-\frac{1}{\beta }\log\left[Z\right]\rangle .$$

We may safely assume that $\langle \tilde{E}(q)\rangle =\langle E(q)\rangle$ so using the definition of the Helmholtz free energy, $A\equiv -\frac{1}{\beta }\log[Z]$,

(A29) $$A\left[\tilde{\eta_{0}}(q)\right]>A\left[\eta_{0}(q)\right],\;\tilde{\eta_{0}}(q)\neq \eta_{0}(q).$$

### A1.4. Applying linear functor to silvera-goldman

The matrix which was ultimately used to transform the Silvera-Goldman potential was obtained by fitting 1000 random potentials with *B* = 50 basis functions in the appropriate *Q* regime. The Silvera-Goldman potential has the form,

(A30) $$V(r)=\exp\left[\alpha -\delta r-\gamma r^{2}\right]-\left(\frac{C_{6}}{r^{6}}+\frac{C_{8}}{r^{8}}+\frac{C_{10}}{r^{10}}\right)f_{c}(r)+\frac{C_{9}}{r^{9}}f_{c}(r)$$

where

(A31) $$f_{c}(r)=\{\begin{array}{ll} e^{-{\left(r_{c}/r-1\right)}^{2}}, & \text{if }r\leq r_{c}\\ 1, & \text{otherwise}. \end{array}$$

Parameters for the Silvera-Goldman potential are provided in Table A1 (Silvera and Goldman, 1978).

**Table A1:** **Parameters of the Silvera-Goldman potential (Silvera and Goldman, 1978)**.

**Parameter**	**Value (atomic units)**
α	1.713
δ	1.5671
γ	0.00993
*C*_6_	12.14
*C*_8_	215.2
*C*_9_	143.1
*C*_10_	4813.9
*r*_*c*_	8.321

Exponential functions cannot be easily represented in a polynomial basis and the Silvera-Goldman potential diverges exponential as *r* approaches zero. Accordingly, we fit the potential only in the physically relevant region of *r* > 4 Bohr. We matched the slope of the potential at *r* = 4 Bohr and extend the potential as a straight line in the region 0 < *r* < 4 Bohr. We choose to fit the potential out to *r* = 24 Bohr but imposed a standard cutoff after the fact at *r* = 20 Bohr as the potential is clearly flat by this point. We simulated *para*-hydrogen at 14K and 25K. At 25K, the thermal de Broglie wavelength is 4.6 Bohr; thus, a cutoff distance of 20 Bohr gives *Q* = 0.23. At 14K, the thermal de Broglie wavelength is 6.2 Bohr and *Q* = 0.31. Based on statistics collected from 10,000 random potentials generated with these *Q* values, in both of these regimes, the classical free energy is more accurate than the  predicted free energy less than 1% of the time.
